# Interdigital Squamous Cell Carcinoma of the Toe Mimicking Infection: Detection of Human Papillomavirus (HPV) Type 6 in a 78-Year-Old Farmer

**DOI:** 10.7759/cureus.108157

**Published:** 2026-05-03

**Authors:** Israa A Serhal, Reem Al Makari, Ali Kallas, Ghenwa K El Dakdoukii

**Affiliations:** 1 Internal Medicine, Hammoud University Medical Center, Sayda, LBN; 2 Internal Medicine, Lebanese University, Beirut, LBN; 3 Infectious Disease, Hammoud University Medical Center, Sayda, LBN; 4 Internal Medicine, Beirut Arab University, Beirut, LBN

**Keywords:** chronic foot lesion, elderly female farmer, human papillomavirus, squamous cell carcinoma, surgical amputation

## Abstract

Human papillomavirus (HPV) is a non-enveloped, double-stranded DNA virus associated with a wide range of benign and malignant lesions affecting mucocutaneous surfaces. Although the oncogenic role of HPV in anogenital and oropharyngeal cancers is well-established, HPV-associated squamous cell carcinoma (SCC) arising in the extremities, particularly the toes, remains exceedingly rare.

We report the case of a 78-year-old female farmer who presented with a persistent lesion between the fourth and fifth toes of her right foot, present for more than 1 month. Physical examination revealed a mildly purulent, horn-like lesion in the interdigital space. Magnetic resonance imaging (MRI) demonstrated a 37 × 17 mm enhancing soft tissue mass suggestive of an inflammatory phlegmon with possible underlying osteomyelitis. Given the clinical and radiological findings, surgical amputation with wide debridement was performed. Histopathological examination revealed a well-differentiated SCC extending to, but not invading, the underlying bone. Surgical margins were negative. HPV DNA typing and cytologic evaluation confirmed the detection of HPV type 6, a low-risk subtype. This case highlights the importance of considering malignancy in chronic, wart-like lesions of the foot, especially in patients with occupational exposure. HPV-associated verrucous SCC, although rare, should be recognized to ensure accurate diagnosis and appropriate surgical management.

## Introduction

Human papillomavirus (HPV) is a non-enveloped, double-stranded DNA virus implicated in a wide spectrum of benign and malignant lesions across mucocutaneous sites. Individuals with persistent infection or multiple sexual partners are at significantly increased risk of acquiring additional subtypes. Clinical manifestations may be overt, presenting as visible lesions, or latent, detectable only through viral DNA testing [1-3]. More than 180 subtypes have been identified. Cutaneous warts of the hands and feet, such as verruca vulgaris and verruca plantaris, are most frequently associated with HPV types 1, 2, 4, 27, and 57. Anogenital warts (condyloma acuminatum) are predominantly caused by HPV types 6 and 11, which are categorized as low-risk; these subtypes are also implicated in both juvenile and adult-onset recurrent respiratory papillomatosis [4]. Digital and subungual squamous cell carcinomas (SCCs) represent a distinct subset of HPV-associated cutaneous malignancies that may mimic benign conditions and frequently present as chronic, treatment-resistant verrucous lesions of the nail unit. This often leads to delayed diagnosis. High-risk HPV types, particularly HPV-16, are the most commonly detected subtypes [5]. While HPV is well-established in anogenital and oropharyngeal malignancies, HPV-associated SCC of the extremities, especially the toes, remains exceedingly rare. Notably, most reported cases involve high-risk HPV subtypes, whereas the pathogenic role of low-risk types, such as HPV-6, in extremity-associated SCC remains poorly understood. Herein, we present a rare case of HPV-associated SCC of the foot, highlighting its diagnostic challenges and therapeutic implications.

## Case presentation

**Figure 1:**
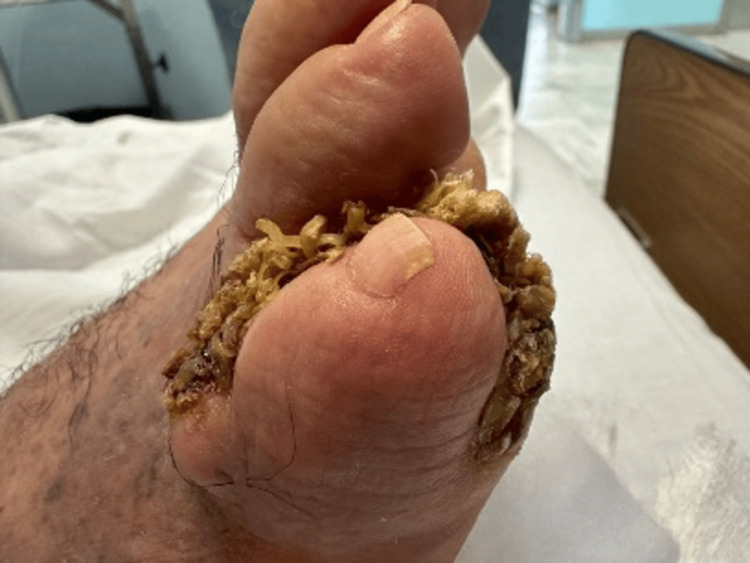
Marked hyperkeratosis between the fourth and fifth toes of the patient's right foot

A 78-year-old, non-smoker female patient, with no significant past medical history, presented with a painless lesion between the fourth and fifth toes of her right foot, persisting for over a month. She denied fever, chills, trauma, or other systemic symptoms and reported no prior history of cutaneous or anogenital warts. The patient reported frequent use of open or non-protective footwear, with prolonged exposure to a moist environment during farming activities. Physical examination revealed a brown, thick, exophytic, verrucous lesion with a hyperkeratotic (horn-like) appearance in the interdigital space between the fourth and fifth toes of the right foot, measuring approximately 4.5 × 1.8 cm. No ulceration, bleeding, induration, or regional lymphadenopathy was noted (Figure 1).

A swab culture was taken, and the patient was started on empirical antibiotics. Due to the suspicion of deep infection, an MRI was ordered to rule out abscess collection or osteomyelitis.

**Figure 2:**
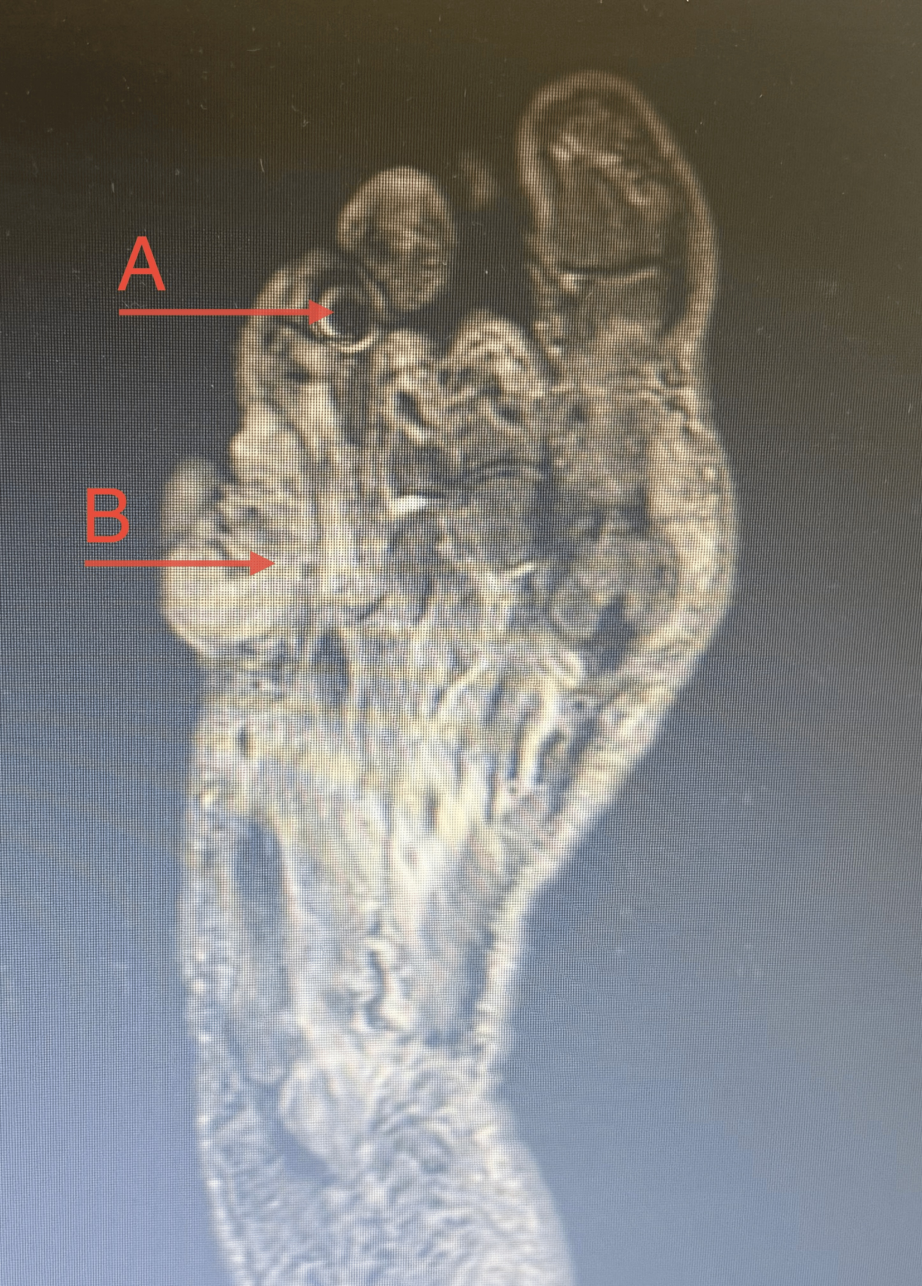
Coronal short tau inversion recovery (STIR) MRI of the lower limb demonstrating a metallic foreign body associated with surrounding phlegmon A: Metallic inclusion B: 37 × 17 mm, enhancing soft tissue lesion in the interdigital space, involving the subcutaneous tissues

MRI showed a 37 × 17 mm, enhancing soft tissue lesion in the interdigital space, involving the subcutaneous tissues. The features were suggestive of an inflammatory phlegmon with possible osteomyelitis. Metallic artifacts were noted, likely related to environmental exposure (Figure 2).

Given the findings, surgical amputation with wide debridement was performed, and intraoperative specimens were sent for pathology and culture. The preoperative swab culture subsequently revealed moderate growth of *Morganella morganii*, while postoperative bone culture was negative, ruling out bone involvement.

**Figure 3:**
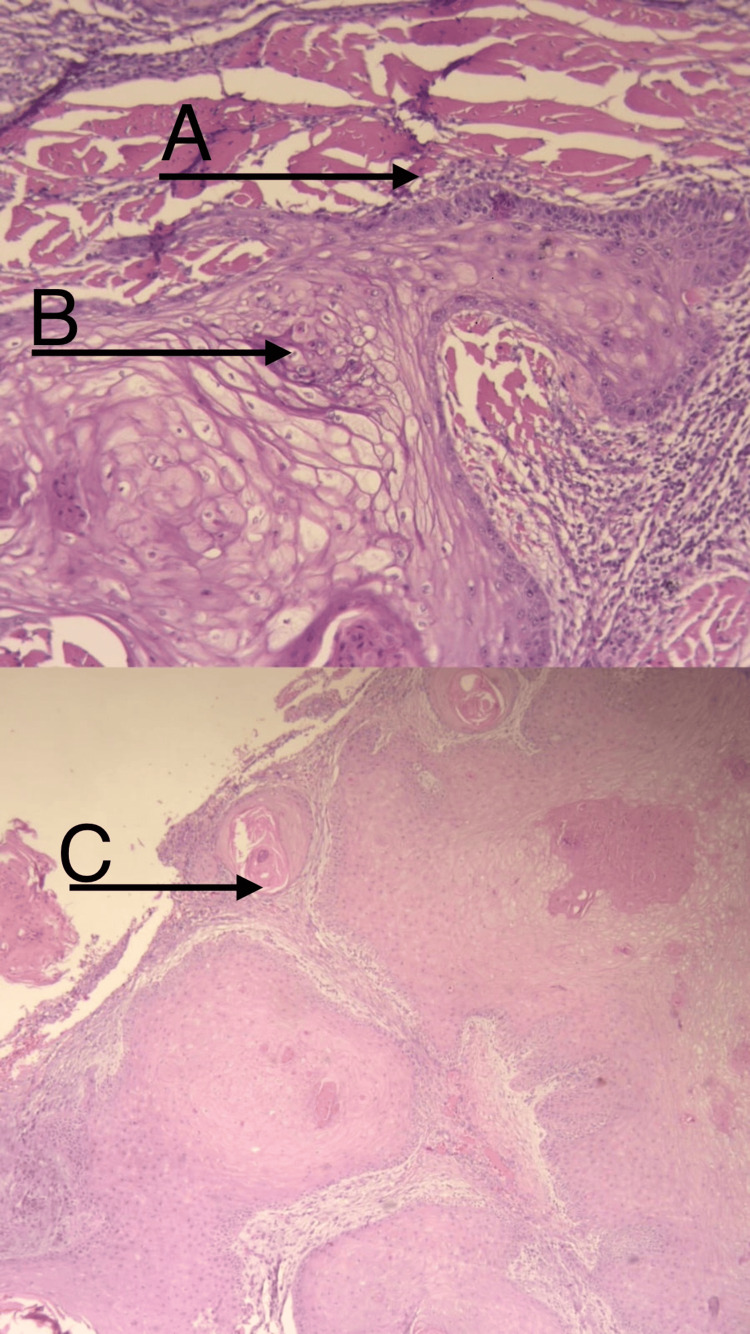
Histopathological features of SCC with koilocytosis suggestive of HPV-associated changes A: SCC invading the reticular dermis with extension into the underlying skeletal muscle. The depth of invasion measures 32 mm. B: Koilocytosis consistent with HPV-associated changes. C: Concentric, whorled layers of keratinized squamous cells. HPV: human papillomavirus; SCC: squamous cell carcinoma

Histological examination confirmed a well-differentiated SCC extending into the reticular dermis and reaching the underlying bone, without evidence of bony invasion. Surgical margins were negative (Figure 3). The clustered verrucous morphology of the lesion raised suspicion for a viral etiology, particularly HPV.

Due to the lesion’s wart-like appearance, chronicity, and the presence of koilocytosis, an HPV panel was performed. HPV DNA typing and cytologic analysis confirmed the presence of HPV-6, a low-risk strain. The assay targeted the E6 and E7 oncogenes of HPV. However, p16 immunohistochemistry and quantitative PCR analyses were not performed due to financial constraints (Table 1).

**Table 1:** HPV PCR result PCR: polymerase chain reaction, HPV: human papillomavirus, FFPE: formalin-fixed, paraffin-embedded PCR detects the following HPV genotypes: High-risk (16, 18, 26, 31, 33, 35, 39, 44, 45, 51, 52, 53, 56, 58, 59, 66, 68, 73, 82) HPV types Low-risk (6, 11) HPV types The detection limit is 50 copies/reaction.

TEST	SPECIMEN	RESULT
Human papillomavirus genotyping (real-time PCR)	FFPE	HPV-6 (low risk detected)

The patient was followed for 12 months postoperatively. She received ciprofloxacin and clindamycin for the management of osteomyelitis, as all surgical margin biopsies were negative for malignancy. An oncology consultation confirmed that no adjuvant therapy was indicated. During follow-up, the patient remained clinically stable, with a single episode of osteomyelitis recurrence requiring hospitalization. She was treated with piperacillin-tazobactam and teicoplanin starting on April 5, 2025 (Figure 3). No further recurrences have been documented since.

**Figure 4:**
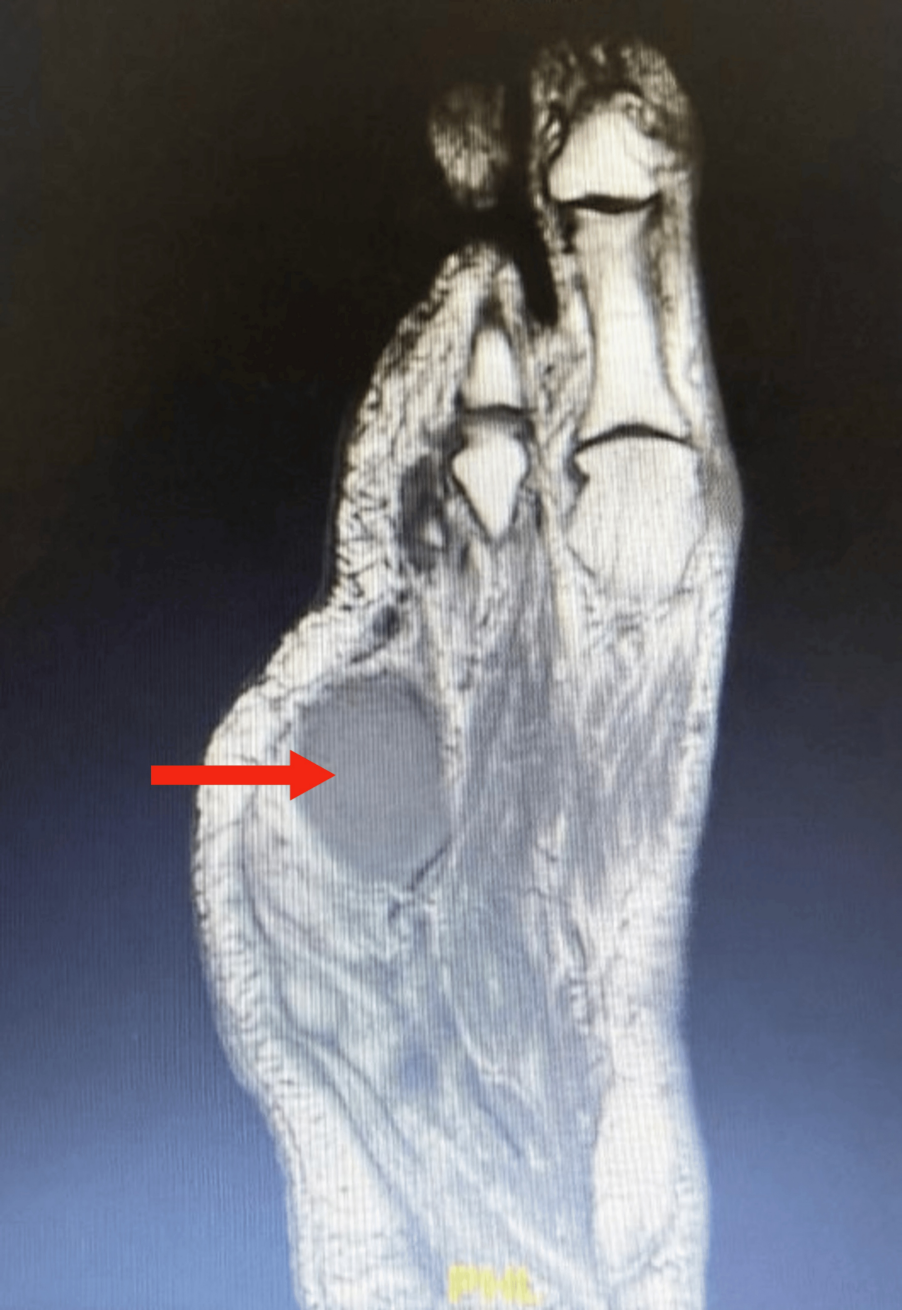
Sagittal T1-weighted MRI of the foot post amputation Large abscess with fluid collection underneath the fourth and fifth metatarsal amputation sites measuring 5.3 cm x 4 cm x 2.5 cm

## Discussion

HPV infection is most often asymptomatic and may persist undetected in the majority of cases. Low-risk HPV genotypes are usually associated with benign proliferative lesions, such as plantar and common cutaneous warts, anogenital warts, and recurrent respiratory papillomatosis. In contrast, high-risk genotypes are strongly implicated in the development of cervical intraepithelial neoplasia (CIN) and malignant transformation at multiple anatomic sites, including the cervix, vagina, vulva, anus, penis, and oropharynx [6]. The oncogenic potential of high-risk HPV is primarily mediated by the viral oncoproteins, E6 and E7. E6 promotes degradation of the tumor suppressor protein p53, thereby impairing apoptosis and DNA repair, while E7 binds to and inactivates the retinoblastoma protein (pRb), leading to deregulated cell-cycle progression. Together, these molecular events create genomic instability, enhance cellular proliferation, and facilitate the accumulation of secondary mutations, ultimately driving malignancy [7].

Epidemiological data highlight the significant contribution of persistent high-risk HPV infection to cancer burden worldwide, with studies attributing approximately 90% of cervical and anal cancers, 70% of vulvar and vaginal cancers, 60% of penile cancers, and 70% of oropharyngeal cancers to these viral subtypes [8]. Importantly, while persistent infection with oncogenic HPV is a necessary condition for carcinogenesis, additional cofactors, such as tobacco exposure, immunosuppression, ultraviolet radiation (in cutaneous disease), and nutritional deficiencies, further influence malignant transformation. Historically, the involvement of alpha-HPV infection in the pathogenesis of SCCs and SCC in situ of the feet was considered unlikely. Instead, alternative predisposing factors, such as genetic susceptibility, radiation exposure, chemical carcinogens, trauma, or chronic tissue irritation and inflammation, were thought to be of greater significance [9]. However, a recent literature review analyzing 36 cases of subungual SCC of the toes demonstrated HPV detection in 19.5% of cases [10]. Several of these lesions harbored high-risk HPV subtypes other than HPV16, including HPV58, HPV56, and HPV45 [11-14], whereas low-risk subtypes, such as HPV-6, have not been reported in this context. Although HPV 6 is traditionally classified as a low-risk subtype with limited oncogenic potential, its presence in malignant lesions may be explained by several mechanisms. Chronic infection in sites subjected to repeated trauma or inflammation, such as the nail unit or interdigital spaces, may promote malignant transformation through sustained epithelial proliferation. In our case, the presence of a metallic inclusion on MRI suggests a potential source of chronic inflammation, which may have contributed to tumor development. Additionally, co-factors such as local immunosuppression or co-infection with other HPV subtypes may facilitate oncogenic progression. Rare cases of malignant transformation associated with low-risk HPV types have been described, suggesting that their role, while uncommon, cannot be entirely excluded. In certain instances, these HPV genotypes were shown to be integrated into the host chromosomal DNA, as evidenced by in-situ hybridization, thereby supporting a pathogenic role for HPV in these tumors [12,13,15].

Histopathological examination demonstrates features suggestive of an HPV-related cytopathic effect, including koilocytosis, alongside the architectural and cytologic features of SCC. In this case, further studies, such as p16 immunohistochemistry and quantitative PCR, were not performed, but may represent useful additional tools in future cases to further support the association between HPV and tumorigenesis by serving as surrogate markers of viral oncogenic activity and viral load, respectively, particularly when integrated with histopathological findings.

To date, three comparable cases of interdigital SCC has been reported, but no one was with a low-risk strain like in our case: a 46-year-old Japanese woman with a history of synchronous invasive vulvar and cervical carcinomas, who developed polydactylous SCC in situ involving the fourth interdigital space of the left foot, as well as lesions on the left thumb, right index finger, and both middle fingers. HPV testing confirmed infection with the high-risk subtype HPV58 [12]. A 78-year-old Caucasian woman presented with a gradually enlarging verrucous plaque in the fourth interdigital space of the right foot, and a 31-year-old Caucasian woman presented with the total destruction of the nail apparatus of the right hallux. Both were found to have HPV-16-associated SCC [11-15]. This case supports the expanding understanding of cutaneous HPV as an oncogenic agent, even in immunocompetent elderly individuals (Table 2).

**Table 2:** Summary of reported cases of HPV-associated SCC Three comparable cases of interdigital SCC have been reported, all linked to high-risk HPV subtypes (HPV58, HPV16), whereas our case uniquely involves a low-risk strain, highlighting the potential oncogenic role of cutaneous HPV. SCC: squamous cell carcinoma, HPV: human papillomavirus

Case and gender	Age	Location	Diagnosis	HPV strain	Treatment
Case 1: Woman	46 yo	4th interdigital space of the left foot, left thumb, right index finger, and both middle fingers [12]	Polydactylous SCCs in situ	HPV 58	-
Case 2: Woman	78 yo	4th interdigital space of the right foot [10]	SCC in situ with focal areas of invasive SCC	HPV 16	Surgical excision of the lesion, with a margin of 5 mm, followed by coverage with a split-thickness skin graft
Case 3: Woman	31 yo	Nail apparatus of the right hallux [10]	SCC in situ	HPV 16	Surgical excision of the lesion, with a margin of 5 mm, followed by wound healing by secondary intention

Clinical relevance

The unusual location and indolent nature of this lesion initially suggested a non-malignant process. However, chronicity, resistance to treatment, and characteristic appearance justified further investigation. In similar presentations, clinicians should maintain a high index of suspicion for viral oncogenesis, even in distal extremities. Early biopsy should be considered in lesions that persist, recur, or fail to respond to standard therapy, regardless of their seemingly benign clinical behavior.

## Conclusions

This case underscores the necessity of considering HPV-related SCC in atypical dermatologic lesions of the toes, especially in patients with chronic exposure to environmental trauma. Early biopsy and HPV testing are crucial for accurate diagnosis and management. While HPV-6 detection is unusual in the context of invasive SCC, it highlights the need for further investigation into the potential role of low-risk HPV subtypes in cutaneous carcinogenesis beyond traditional mucosal sites.
